# Job vacancy at ECDC

**DOI:** 10.2807/1560-7917.ES.2022.27.18.220505v

**Published:** 2022-05-05

**Authors:** Eurosurveillance editorial team

**Affiliations:** 1 European Centre for Disease Prevention and Control (ECDC), Stockholm, Sweden

The European Centre for Disease Prevention and Control (ECDC) invites applications for the following position:

 

- **Principal Expert Communicable Diseases Prevention and Control**
  The application deadline expires on 30 May 2022.

 

For more information, please visit the ECDC website: 

https://ecdc.europa.eu/en/work-us/vacancies?f%5B0%5D=deadline_date%3A1

